# Excision of Large Scalp Arteriovenous Malformations with Aesthetic Scalp Reconstruction

**DOI:** 10.29252/wjps.9.3.302

**Published:** 2020-09

**Authors:** Anupama Singh, Ankur Bhatnagar, Vivek Singh

**Affiliations:** 1 Department of Plastic Surgery, Sanjay Gandhi Post-Graduate Institute of Medical Sciences (SGPGIMS), Lucknow, India;; 2 Department of Radiodiagnosis Sanjay Gandhi Post-Graduate Institute of Medical Sciences (SGPGIMS), Lucknow, India

**Keywords:** Embolization, Tissue expansion, Arteriovenous malformation, Scalp

## Abstract

**BACKGROUND:**

Scalp arteriovenous malformations (SAVMs) are seen in young individuals and skin involvement is common in large SAVMs. They are commonly seen in younger age group too. Pre-operative embolization followed by surgical excision and hair bearing scalp reconstruction with tissue expansion are the treatment of choice. Therefore, proper selection of tissue expander for reconstruction of hair bearing scalp, seems essential. This study evaluated excision of large SAVMs with aesthetic scalp reconstruction.

**METHODS:**

We described management of 10 patients of large SAVMs with cutaneous involvement. All patients underwent pre-op embolization followed by surgical excision and hair bearing scalp reconstruction with tissue expansion.

**RESULTS:**

All cases of large SAVMs healed well with minor complications.

**CONCLUSION:**

While complete surgical excision with extirpation of the nidus is considered as the gold standard treatment, aesthetic hair bearing scalp restoration is also of paramount importance for the patient. This is done by using scalp tissue expansion after proper selection of the expander.

## INTRODUCTION

Arteriovenous Malformations (AVMs) are high flow vascular malformations found all over the body.^1^ Scalp has the thickest layer of skin in the body (3-8 mm)^2^ and scalp arteriovenous malformations (SAVMs) account for 8% of all AVMs.^1^ SAVMs mostly originate in the subcutaneous layer of scalp which contain the vessels and nerves and rarely has intracranial extension.^2^ Skin involvement is fairly common especially in large AVMs as long standing cases and recurrent lesions due to inadequate excision. Most of cases are congenital and present in the 2^nd^ or 3^rd^ decade. Unlike truncal or extremity ones, SAVMs are detected early due to superficial location and cosmetic deformity. Patients present as a throbbing pulsatile mass with headache and cosmetic deformity. Rarely patients present with recurrent bleeding or haemorrhage following trivial trauma.^1^^,^^2^

Multimodality treatment with complete surgical excision is the treatment of choice. Since most of the patients are young individuals, restoration of hair bearing scalp is of paramount importance, to allow their successful re-integration into the society. In massive SAVM cases, when scalp preservation is not possible, reconstruction of hair bearing scalp becomes very important. Hence, multispecialty and multimodality treatment is essential to provide natural and aesthetic hair bearing scalp restoration following excision.^1^^,^^2^ This study evaluated excision of large SAVMs with aesthetic scalp reconstruction.

## MATERIALS AND METHODS

This study retrospectively included 10 patients of diagnosed extracranial large SAVMs having extensive cutaneous involvement, where primary skin closure was not possible post-excision. The study was approved by institute ethics committee as per standard procedure (IEC Code 2020-145-IP-EXP-21). The study was cleared by Institute Ethics Committee of Sanjay Gandhi Post Graduate Institute of medical Sciences, Lucknow. India. All patients underwent staged aesthetic scalp restoration post-excision using tissue expanders. Clinical data, investigations, details of surgical procedures, and post-operative follow up was noted for all the patients. Each patient underwent preoperative screening Doppler followed by digital subtraction angiography (DSA) to map the feeding vessels and MR angiography to ascertain the extent of lesion and rule out any intra cranial extension.

**Fig. 1:**
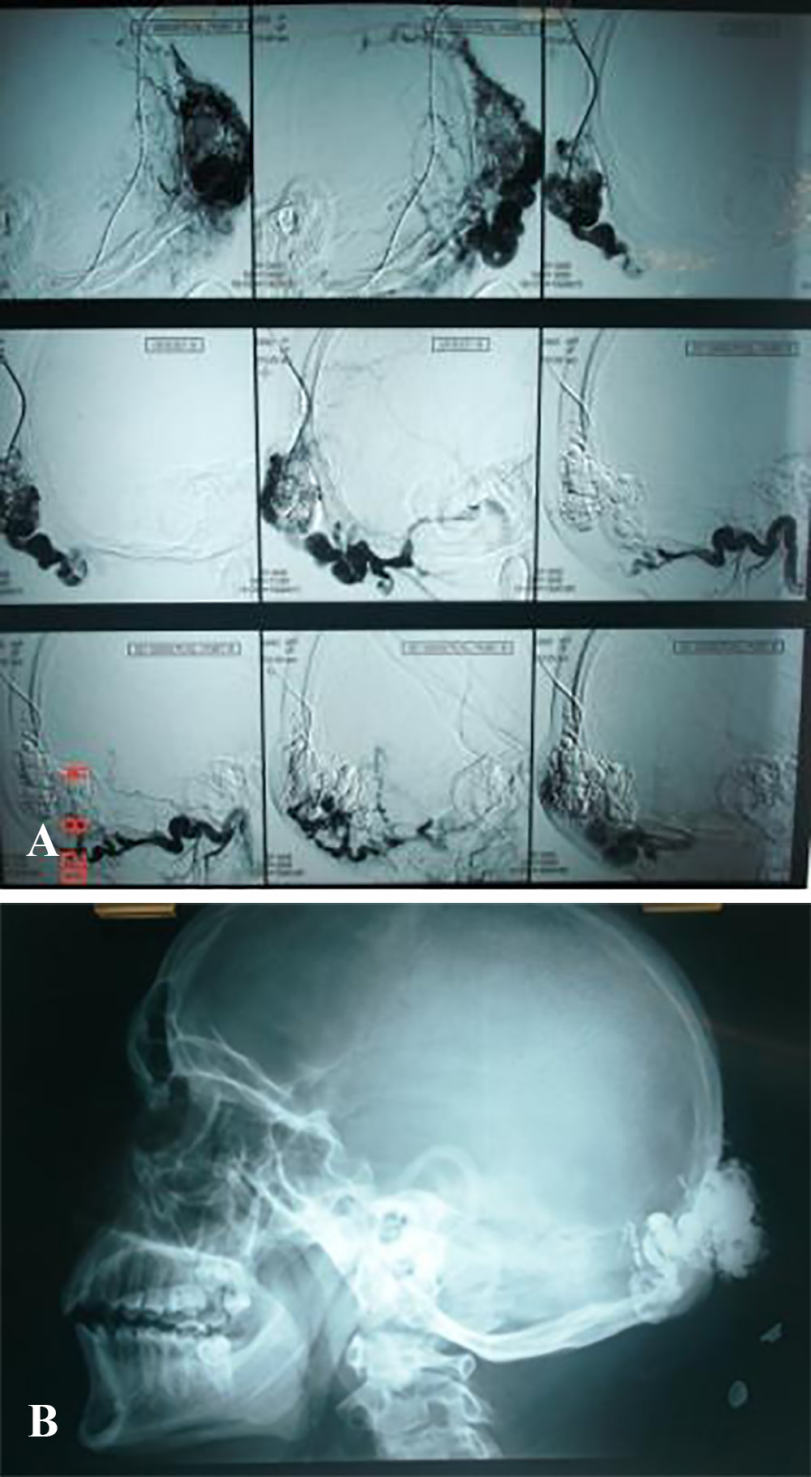
**A: **Glue embolization of nidus using direct puncture. **B:** X-ray of skull with nidus embolized prior to surgical excision

**Fig. 2:**
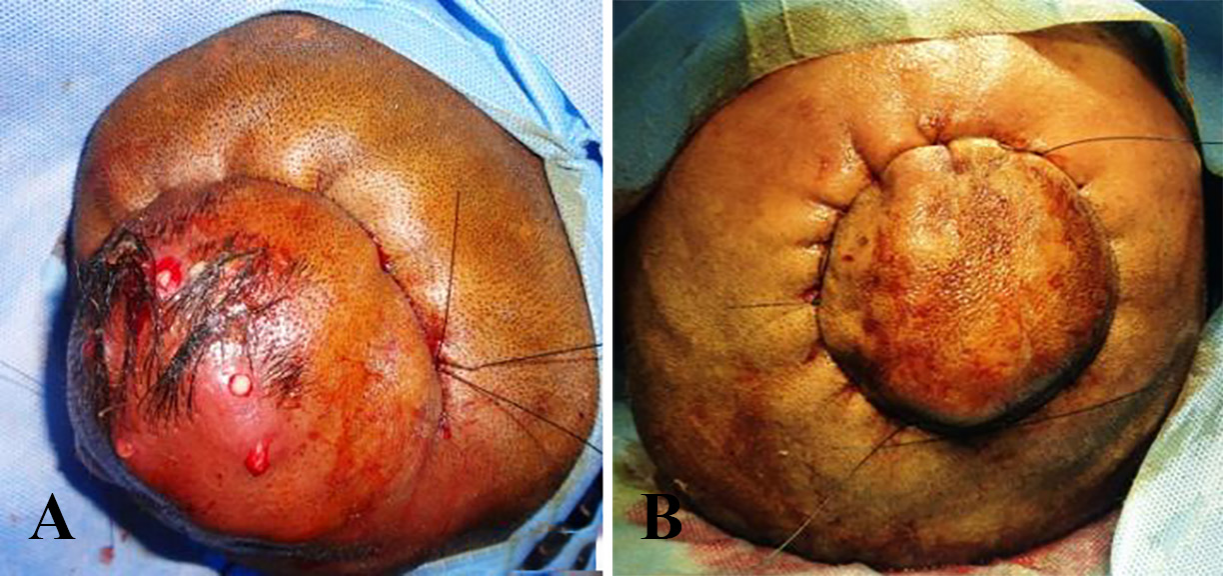
**A, B: **Interlocking haemostatic suture

After assessment of general fitness, all patients underwent glue embolization in the department of intervention radiology. Embolization involved direct puncture of the nidus under USG control (Figure 1A and 1B). Two to three weeks post-embolization, wide local excision of the lesion was performed under GA, where in the entire lesion with the involved overlying skin was excised. The underlying periosteum was preserved as it was not generally evolved. To control intraoperative haemorrhage, the entire incision line was infiltrated with 1 in 2000000 of diluted epinephrine solution. Interlocking haemostatic sutures were applied around the entire lesion keeping a margin of 1 cm (Figure 2A and 2B).

**Fig. 3:**
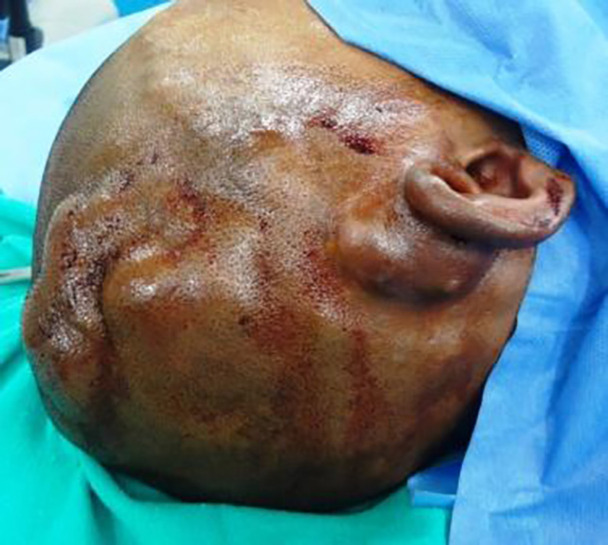
Extensive SAVMs with skin involvement post-embolization

**Fig. 4:**
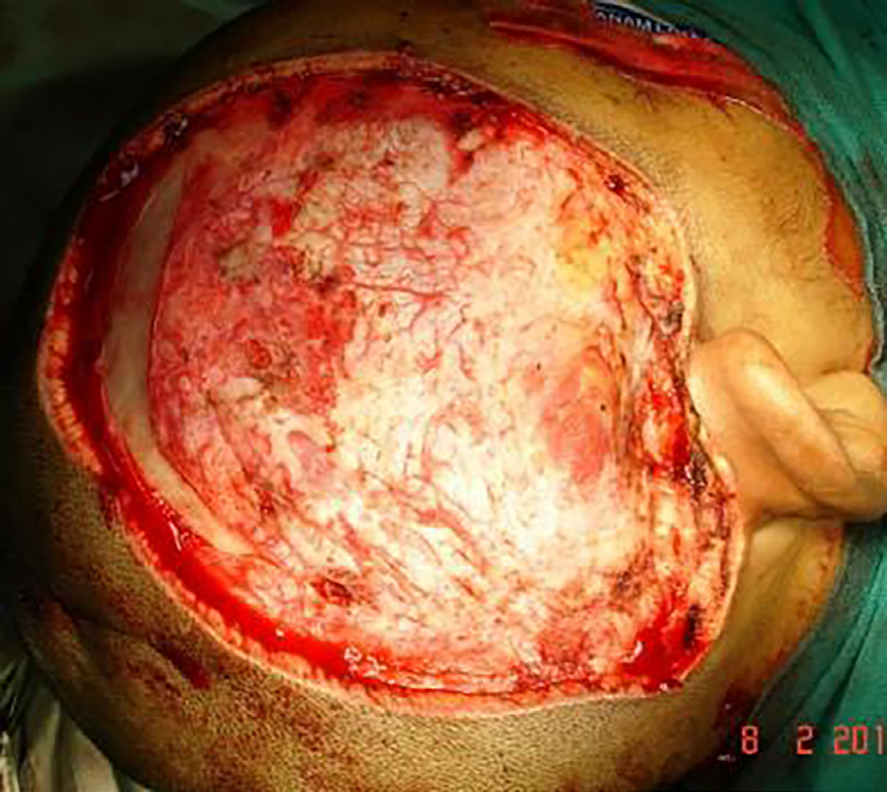
Post-excision with preserved pericranium

**Fig. 5:**
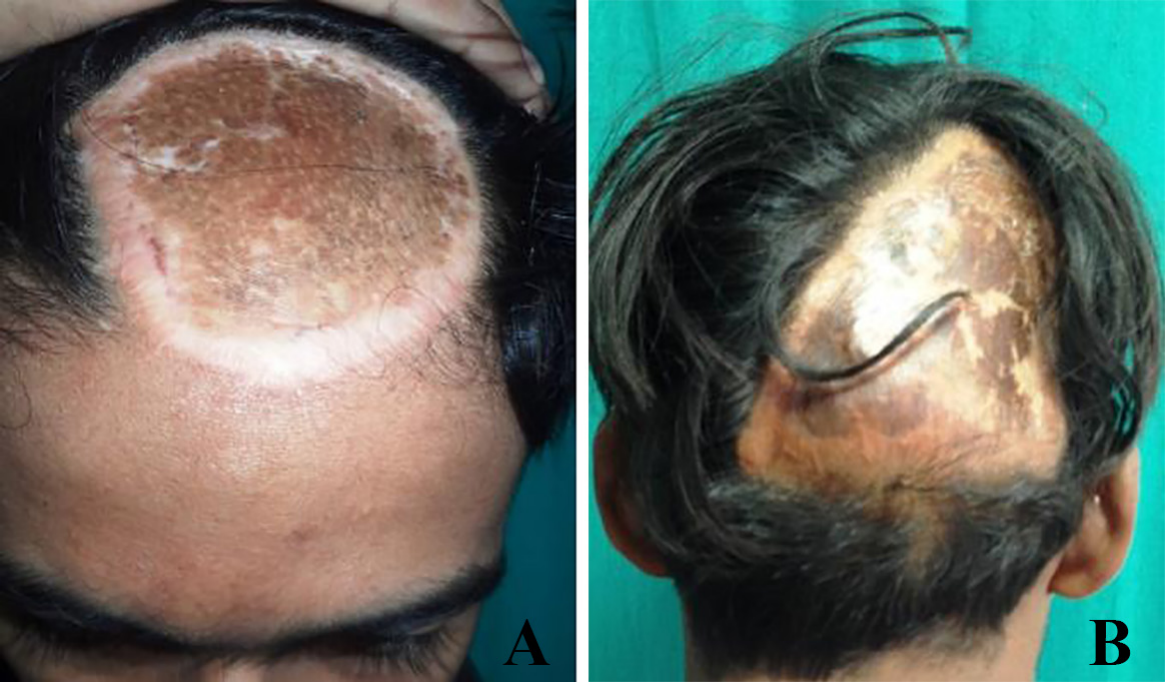
**A, B: **Well settled SSG over pericranium

Once entire lesion was excised, interlocking sutures were removed and haemostasis was achieved (Figure 3 and 4). The residual raw area was then resurfaced with thick split thickness skin graft harvested from the thigh. Three to 5 weeks post-surgery, stable graft takes were achieved and all dressings were discontinued (Figure 5A and 5B). All patients were kept in follow up for 6 months, when a repeat Doppler study was performed to rule out any recurrence. All patients were then given an option for a staged hair bearing scalp reconstruction using tissue expanders. 

**Fig. 6:**
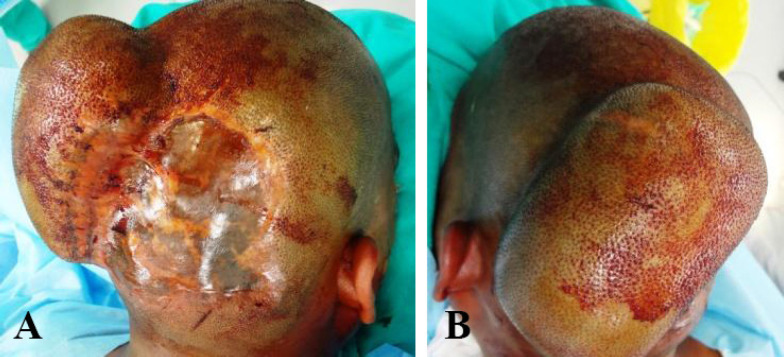
**A, B: **Uninvolved expanded scalp skin with rectangular tissue expander

In stage I, an adequate sized tissue expander was placed in the subgaleal plane just adjacent to the skin grafted region. Care was taken to incorporate at least one malor scalp artery, most commonly superficial temporal artery of the opposite side in the expanded skin territory. Two to 3 weeks following expander placement, serial weekly expansion was started with distilled water (Figure 6A and 6B). On average, 30 to 35 mL of fluid was injected per week in patients with tissue blanching or pain being the endpoint. 

**Fig. 7:**
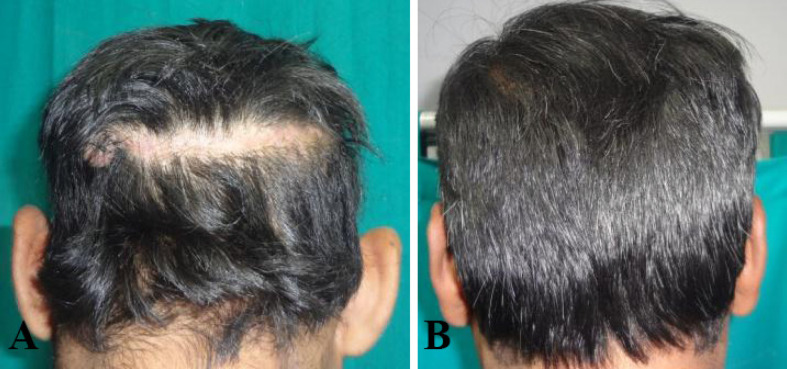
**A: **Good scalp repair with minimal scar.** B: **Long term results with hair growth camouflaging the residual scar

Expander removal was performed at least 3 weeks after desired expander volume had been achieved. After removal, the extra-skin was then used to cover the calvarium as an advancement flap after excision of the previous skin graft (Figure 7A). Residual unexpanded scalp skin was also raised as flap and mobilized. In the expanded flap and also the residual non-expanded scalp skin multiple galeal cuts were made perpendicular to the line of advancement to allow tension free closure. Trichophytic closure was done to reduce the residual scar and provide aesthetic scalp reconstruction as described before^2^ (Figure 7B).

## RESULTS

All 10 cases had high flow AVMs with extensive skin involvement (Table 1). In such cases primary scalp closure or a rotation flap was not possible. All patients were young individuals in the second or third decade of life including 8 males and 2 females. Lesions ranged from 90 cm^2 ^to 130 cm^2^, while post-excision skin defects ranged from 40 to 80 cm^2^. Average blood loss per case post-excision was less than 100 mL. In our study, rectangular expanders were used in 9 cases, while crescentic was used in one case.

**Table 1:** Patients with high flow AVMs and extensive skin involvement

**No**	**Gender**	**Age**	**Site of lesion**	**Size of lesion cm** ^2^	**Size of Skin defect cm** ^2^	**Expander volume**	**Shape**	**Remarks**
1	M	22	TP	108	66	500	Rectangle	
2	M	25	TP	120	72	500	Rectangle	
3	M	28	P	100	44	300	Crescentic	Marginal Skin necrosis
4	F	19	FT	90	63	300	Rectangle	
5	M	26	FTP	117	77	500	Rectangle	Marginal Skin necrosis
6	M	28	O	88	45	300	Rectangle	
7	M	17	OP	99	50	500	Rectangle	
8	M	21	OP	121	63	500	Rectangle	Marginal skin necrosis
9	F	20	TP	96	36	300	Rectangle	
10	M	25	OP	88	48	300	Rectangle	

For smaller lesion 300 mL of expanders were used, while for larger ones 500 mL of expanders were applied. In all cases, post-excision graft take was good and patients were dressing free, 3 to 5 weeks after surgery. Minimal graft loss was seen in two patients that healed by secondary intention. Post-expander removal and flap advancement, primary healing was visible in all cases. Three patients had minimal flap loss at the distal edge that healed by secondary intention. In all cases, expander port was placed in the post-auricular region near the mastoid process for easy access during the fluid injection. 

All patients were followed up for at least one year after expander removal to look for recurrence, hair growth and scar widening. On average, the scar widened by 3 mm which was more in patients who had terminal flap compromise. However, none of the patients underwent a repeated excision, as the scar was well hidden by good hair growth in the expanded scalp skin. No patient had recurrence of the lesion during the follow-up.

## DISCUSSION

AVMs are abnormal fistulous connections between feeding arteries and veins without an intervening capillary bed. SAVMs are accounting for 8% of all AVMs and are mostly present in the subcutaneous and cutaneous scalp layer.^1^ They can be present in all scalp regions with temporo-parietal being the commonest, as also evident in our study.^3^ Main source of feeders is from superficial temporal and occipital artery. Persistence of embryonal capillary network in later stages of gestation leads to formation of intercommunicating channel, which results in formation of SAVMs.^3^

Most of cases are congenital and become significantly large enough in the second or third decade of life to merit attention.^3^ Unlike limb or truncal AVMs, SAVMs are detected early due to their superficial position and profound cosmetic deformity.^1^ Clinically, presence of a compressible soft tissue mass, visible expansile pulsations and presence of bruit makes clinical diagnosis easy. Growth of untreated SAVMs is seen during puberty, pregnancy and following trauma. This is attributed to the change in concentration of circulating hormones with increased VEGF leading to neovascularization.^1^ Patients can also present with throbbing pain, headache and even recurrent haemorrhage following trivial trauma. Skin necrosis is seen in patients who have undergone therapeutic embolization without surgical excision.^4^

Diagnosis of SAMVs is confirmed with a Doppler USG, which shows high flow patterns. DSA helps to find out the many feeders to the lesion and also whether intracranial involvement is present or not. DSA can be used to differentiate other vascular lesions like aneurysms and sinus perircanii.^3^ MR angiography helps estimation of the form and extent of the lesion and also any intracranial extension. MR angiography helps planning the incisions before surgery, so that uninvolved major scalp vessels could be preserved. The skin flaps are planned around these vessels for placement of expander and subsequent reconstruction of hair bearing scalp.^3^

Once diagnosis of extracranial SAVMs is confirmed, treatment protocol for the patient has to be decided. In our study, all patients were in schobinger stage I/II^1^ (Table 2); hence, the indication for treatment was mostly cosmetic. Complete surgical excision is the gold standard treatment. Biggest problems in surgical excision are intra-operative haemorrhage and post-operative recurrence. Many times, significant intra-operative haemorrhage is observed that can lead to incomplete excision and recurrence.^4^

**Table 2:** Schobinger classification of arteriovenous malformations

**Stage**	**Features**
1 (Quiescence)	Cutaneous blush/warmth
2 (Expansion)	Bruit, audible pulsations, expanding lesion
3 (Destruction)	Pain, ulceration, bleeding, infection
4 (Decompensation)	Cardiac failure

Various methods have been used both pre-operatively and intra-operatively to control hemorrhage. Pre-operative glue embolization either through direct nidus puncture or through micro catheters have been used with great success. Nidus embolization is important for long term control. Isolated feeder embolization, does reduce vascularity of the lesion, but makes the AVMs prone to paradoxical increase in size due to relative ischemia in the functional nidus, which causes the release of pro-angiogenic factors. This is akin to physical ligation of arterial feeders, which may initially decrease the vascularity of the lesion, but relative ischemia of the nidus paradoxically increases the growth of AVMs, if not excised.^1^^,^^4^

Embolization has few relative drawbacks. Firstly, it is expensive and secondly, it will require a trained interventional radiologist, which may not be possible in various resource limited settings.^4^ Endovascular embolization without surgical excision is prone for recurrence. Moreover, such patients have high incidence of skin necrosis with development of chronic sinus and ulceration.^1^ This may be due to the fact that embolization alone produces tissue ischemia, which causes necrosis of the cutaneous tissue and leads to ulceration and sinus formation. Moreover, recurrence of lesion may occur few months down the line, if entire nidus is not excised.^3^

Endovascular embolization can be curative in very small SAVMs and fistulas.^3^ It may also be indicated as treatment for extremely large SAVMs in patients with co-morbidities, where surgical treatment is contraindicated.^1^ In our study, pre-operative glue embolization with direct nidus puncture under USG guidance was done. This was supplemented with angiographic feeder embolization, when response from direct puncture was found to be inadequate. In our study, average blood loss was less than 100 mL during excision with no need for intra- or post-operative blood transfusion.

Another method for hemorrhage control is the temporary ligation of external carotid artery (ECA) during excision.^4^ This should theoretically decrease haemorrhage, but due to extensive collateral circulation in the scalp and flow from opposite ECA, complete bloodless field is not possible. However, if angiographic embolization is not available, then ECA ligation is a viable option. Other methods which our study used to decrease blood loss were infiltration of the incision margins with diluted epinephrine solution and use of interlocking cutaneous sutures. 

In our study, the entire nidus with involved skin was excised under hemostatic control. One of the major advantages of pre-operative embolization and use of hemostatic sutures was to have a virtually bloodless intraoperative field during excision. In our study, we could preserve the underlying pericranium as sub galeal plane was easy to dissect in a blood less field. In other studies where pre-operative embolization was not used.^2^^,^^4^^,^^8^

Several authors have raised skin flaps with pericranium, excising non-involved tissue along with the lesion.^2^^,^^4^^,^^8^ Once calvarial bone is exposed, cover has to be done with local transposition of scalp flaps and with skin grafting to the flap donor area. This involves manipulation of the un-involved scalp skin that makes subsequent hair bearing scalp reconstruction difficult. In our study, due to intact pericranium, skin graft was placed over the defect and tissue expanders were placed in the virgin normal adjacent scalp skin in subsequent stages, thus minimizing expander related complications like wound dehiscence, implant exposure, scar expansion and residual alopecia. 

Once no recurrence is observed, aesthetic hair bearing scalp reconstruction especially for young patients is a must. No other tissue in the human body mimics hair bearing scalp,^5^ and providing a near normal hair bearing scalp cover, should be final goal as most of SAVM patients are young individuals with major aesthetic concerns. Multiple methods are available for hair restoration from primary closure, rotation and advancement flaps, hair transplant, scalp reduction, scalp expansion and even free tissue transfer. By selecting appropriate option based on the anatomical location and size of the defect, aesthetic reconstruction with hairline restoration can be achieved.^6^

Free tissue transfer is generally done for extensive lesions especially oncologic or post-burn alopecia that provides a stable cover, but is unsuitable to provide hair bearing scalp. Hair transplant although common, has poor results in grafted skin, where hair follicle uptake is poor. For good hair restoration, minimum density of 20-25 follicles per cm^2 ^is essential, which in such cases can only be provided by native scalp skin.^7^ Since in all cases, skin defect was > 20 cm^2^, primary closure and local flaps without SSG was not possible. 

Hence, in all cases after placement of SSG in the initial surgery, scalp reconstruction was done using tissue expansion with silicone expanders. Tissue expansion has been a standard treatment for hair restoration in extensive post-burn and cicatrial alopecia. Middle sized scalp defects (less than 50% of total scalp loss), can be reconstructed using this method, while maintaining homogenous good hair density with relatively low complications.^7^ Scalp with its rich blood circulation, thick overlying tissue and rigid skull bone below is an ideal place for expander placement.^5^

Tissue expansion is governed by concepts of biological and mechanical creep that increases mitotic activity and intrinsic extensibility of the native skin.^2^^,^^5^ Same principals were used in our study. Size, shape and placement of implant provide the optimal results with least complications. In our study, in 9 out of 10 cases, rectangular expanders were used as they provided the most efficient expansion.^5^ The width of the defect was noted and an expander which could generate extra skin 2.5 times the width of defect was taken. The volume of such expander was calculated to about 5-7 mL/cm^2 ^of the lesion.^5^

Expander was placed in the sub-galeal plane. The scalp flap for resurfacing of the lesion was planned such that, at least one un-involved artery was preserved in the flap base. This helped in minimizing the post-operative complications and prevented scar expansion. Trichophytic skin closure allows good wound edge approximation providing effective camouflage of incision. However, minimal scar expansion is expected in postoperative phase which can be camouflaged by the good hair growth in the scalp flap.^2^

## CONCLUSION

SAVMs are commonly seen in younger age groups. While complete surgical excision with extirpation of the nidus is the gold standard treatment, aesthetic hair bearing scalp restoration is also of paramount importance for the patient. Pre-operative embolization along with intraoperative hemostatic measures helped achievement of a relatively bloodless field during surgery, enabling total excision of the lesion, preservation of pericranium and avoiding recurrence. Temporary intra-operative ligation of ECA is an inferior, but viable alternative to pre-operative embolization. Tissue expansion is the best option for restoration of hair bearing scalp, when skin defects are large. Proper selection of expander size, volume and correct placement preserving the un-involved arteries in the expanded scalp flap gives long lasting stable hair restoration in these cases.
